# Antimicrobial and Virucidal Potential of Morpholinium-Based Ionic Liquids

**DOI:** 10.3390/ijms24021686

**Published:** 2023-01-14

**Authors:** Jakub Michalski, Julia Sommer, Peter Rossmanith, Anna Syguda, Tomasz Clapa, Patrick Mester

**Affiliations:** 1 Department of Biochemistry and Biotechnology, Poznań University of Life Sciences, Dojazd 11, 60-632 Poznan, Poland; 2 Christian Doppler Laboratory for Monitoring of Microbial Contaminants, Unit for Food Microbiology, Department of Veterinary Public Health and Food Science, University of Veterinary Medicine, 1210 Vienna, Austria; 3 Epitome GmbH, The ICON Vienna, Tower 17, Gertrude-Fröhlich-Sandner-Str. 2–4, 1100 Vienna, Austria; 4 Unit of Food Microbiology, Institute of Food Safety, Food Technology and Veterinary Public Health, Department for Farm Animals and Veterinary Public Health, University of Veterinary Medicine Vienna, Veterinärplatz 1, 1210 Vienna, Austria; 5 Department of Chemical Technology, Poznan University of Technology, Berdychowo 4, 60-965 Poznan, Poland

**Keywords:** ionic liquids, antibacterial agents, antiviral activity, enzyme activity

## Abstract

Witnessed by the ongoing spread of antimicrobial resistant bacteria as well as the recent global pandemic of the SARS-CoV-2 virus, the development of new disinfection strategies is of great importance, and novel substance classes as effective antimicrobials and virucides are urgently needed. Ionic liquids (ILs), low-melting salts, have been already recognized as efficient antimicrobial agents with prospects for antiviral potential. In this study, we examined the antiviral activity of 12 morpholinium based herbicidal ionic liquids with a tripartite test system, including enzyme inhibition tests, virucidal activity determination against five model viruses and activity against five bacterial species. The antimicrobial and enzymatic tests confirmed that the inhibiting activity of ILs corresponds with the number of long alkyl side chains and that [Dec_2_Mor]^+^ based ILs are promising candidates as novel antimicrobials. The virucidal tests showed that ILs antiviral activity depends on the type and structure of the virus, revealing enveloped Phi6 phage as highly susceptible to the ILs action, while the non-enveloped phages PRD1 and MS2 proved completely resistant to ionic liquids. Furthermore, a comparison of results obtained for P100 and P001 phages demonstrated for the first time that the susceptibility of viruses to ionic liquids can be dependent on differences in the phage tail structure.

## 1. Introduction

Ionic liquids (ILs) are a novel class of chemical compounds that have caught the attention of researchers all around the world. Due to their unique properties, which include negligible vapor pressure or non-flammability, they have been included in the “green chemistry” sector [1]. One of the fields where ionic liquids have been successfully applied is the agricultural sector, where the constantly increasing human population and food demand is in need for innovative and highly efficient agrochemicals for various applications. In this context, herbicidal ionic liquids (HILs), where classic herbicides are converted into either anions or cations, and their potential for manipulating properties, such as volatility, toxicity and biodegradability, have been intensively researched [2,3,4]. 

In addition to their application as herbicides, in a recent study, we could demonstrate that morpholinium-based HILs could also have potential application as effective antimicrobials, as some were highly effective against clinical isolates of the opportunistic human pathogen *Pseudomonas aeruginosa* [5]. In this study, we could show that ILs with a [Dec_2_Mor]^+^ cation are more toxic to *P. aeruginosa* strains than ILs with a [DecEtMor]^+^ cation and that all morpholinium-based HILs were decreasing the virulence of the investigated strains. We could recently also show that ILs are influencing at the molecular levels of different and pathogenic isolates of *P. aeruginosa*, which cause problems not only with microorganisms (free-living bacterial cells and also with biofilms), but mostly with human beings [6]. It is known that at the current moment, the chemical structures can be synthesize in an amount abound 10^18^ so the new applications of the ILs still cannot be known [7]. 

These initial findings of high antimicrobial activity warrant further evaluation of these ILs, and for this reason, we investigated the set of morpholinium-based ILs using a tripartite biological test system of diverse complexity, including an enzyme inhibition test, an evaluation of the virucidal activity and bactericidal activity [8]. Enzyme inhibition has already proved to be a good indicator of IL toxicity, and the qPCR-based inhibition test was chosen because it is a highly sensitive method and is based on the well-established PCR reaction. The five viruses (P100, P001, PRD1, Phi6, and MS2) represent suitable surrogates for the most essential virus groups and were previously used in IL studies. The set of Gram-positive and Gram-negative bacteria (*Listeria monocytogenes*, *Salmonella enterica*, *Escherichia coli*, *Lactococcus lactis* and *Pseudomonas syringae*) provides a broad spectrum of different bacterial orders and is anticipated to reveal species-specific differences [8,9,10,11]. In the case of the bactericidal activity of ILs, different mode-of-action was previously described, such as generating reactive oxygen species, influencing membrane permeability, activating the SOS system that is responsible for protecting living organisms, or inhibiting enzyme activity, each of which can determine the general applicability of ILs as antimicrobials [6,9,12,13,14,15]. As in our previous study, only strains of the Gram-negative *P. aeruginosa* were tested, it is crucial to confirm the activity against other species, including also Gram-positive bacteria.

In addition to the application as antimicrobials, ILs have recently also been studied as novel and efficient virucidal agents, and the need for novel virucides has been dramatically highlighted by the recent pandemic. While the SARS-CoV-2 virus is an enveloped virus and thus easier to inactivate, this is not the case for other viruses. While viruses vary widely in their ability to survive outside the host [16,17,18], they generally have a low minimal infective dose [19], and thus, the longer they can remain infectious in the environment, the greater the risk of their spread from environmental sources [20]. The spread of viral infections can be further amplified by unhygienic conditions and overcrowding as exemplified by improperly maintained institutional settings such as children’s daycare centers, hospitals and nursing homes [20,21]. While vaccination against several viral diseases has been remarkably successful [22,23], safe and effective vaccines remain unavailable against many viral infections. As a result, effective decontamination of environmental surfaces and medical devices (e.g., endoscopes) with microbicides plays a vital role in environmental control of viral infections, especially for viruses that are not mainly spread directly via air, food, injections or direct venereal/non-venereal person-to-person contact [24]. These factors together re-emphasize the need for virucides, microbicides (disinfectants and antiseptics), resulting in a permanent loss of virus infectivity after exposure, for controlling and preventing the spread of viral diseases. The activity of a virucide depends upon a number of factors, some inherent to the chemical nature of the microbicides (e.g., concentration, pH, contact time, and relative humidity), some inherent to the conditions on application (e.g., type of surfaces, temperature, and soiling) and some inherent to the structure and genome content of the viral particle [25]. While there are several important classes of virucides, including surface acting agents (e.g., quaternary ammonium compounds, halogens, phenols, oxidizing agents, alcohols, acids, and alkalis), our understanding of the activity and the mechanisms of action of microbicides against viruses remains quite fragmentary [25,26,27]. To make matters worse, recent studies demonstrated the ineffectiveness of commercial sanitizers against viruses, as well as the increasing development of their resistance, emphasizing the need to develop new, more effective virucides, especially against non-enveloped viruses [28,29,30]. 

In this study and based on the results of Cłapa et al. (2021) [5], twelve different morpholinium based HILs were investigated as a promising class of novel biocides and virucides using a tripartite biological test system.

Overall, the application of this tripartite biological test system to investigate the toxicity of HILs offers broad relevance to describe their bactericidal and virucidal potential.

## 2. Results

### 2.1. Enzymatic Toxicity

**Figure 1:**
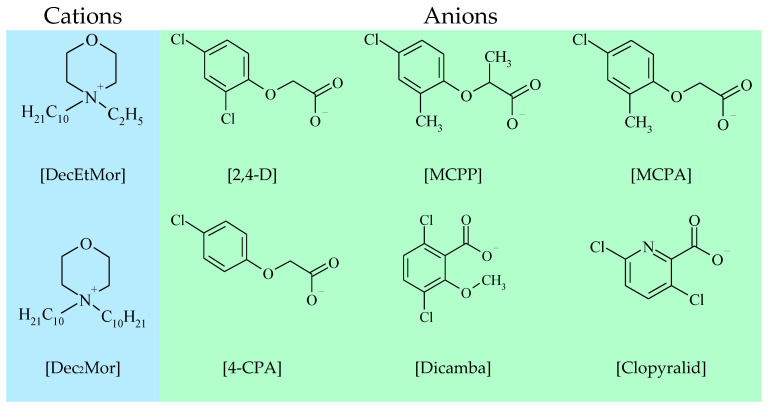
Graphic representation of all cations and anions of morpholinium based HILs investigated in this study.

Due to the increasing interest in ILs biological and antimicrobial characteristics, different enzyme inhibition assays have been established as a simple and fast pre-screening method for the determination of the toxicological potential of ILs [31,32,33,34]. In this study, an established method based on a qPCR assay was used, which has been applied for the determination of the toxic potential of pyrithione-ILs [10], fatty-acid-ILs [9] and for the determination of ILs with special structural motifs, such as increasing side-chain length, anion chaotropicity and others [8]. With this quantitative polymerase inhibition assay, the minimal inhibitory concentration (MIC) as well as the half-maximal effective concentration (EC_50_) of 12 morpholinium based HILs was determined (Figure 1; full list of HILs Table S1). 

**Figure 2:**
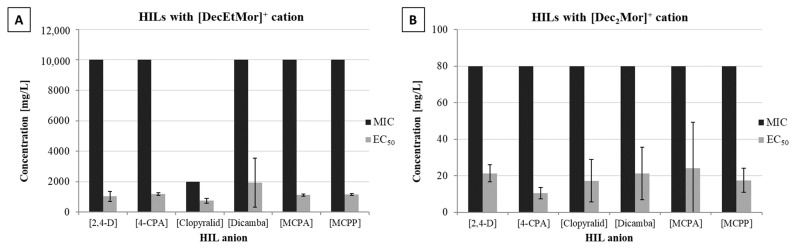
Minimum inhibitory enzyme concentration [mg/L] ± SD (black) and half-maximum effective concentration [mg/L] ± SD (grey) of HILs with either [DecEtMor]^+^ cation (**A**) or [Dec_2_Mor]^+^ cation (**B**). Note the ordinate scale differences between the two panels.

The results in Figure 2A,B and Table S2 show that especially the ILs with a [Dec_2_Mor] cationic core exhibited a significant enzyme inhibition potential with EC_50_ values between 10–24 mg/L and complete inhibition was observed at 80 mg/L for all six ILs. In contrast to the [Dec_2_Mor] ILs, [DecEtMor] ILs exhibited a less pronounced enzyme inhibition potential. Except for the liquid [DecEtMor][Clopyralid] that indicated a MIC of 2000 mg/L, MIC of all other [DecEtMor] ILs was equal to 10,000 mg/L. EC_50_ values of [DecEtMor] ILs were found in a range between 751 and 1947 mg/L.

### 2.2. Antimicrobial Activity

As previously reported, morpholinium-based HILs demonstrated high antimicrobial activity against *P. aeruginosa* [5]. In order to determine if such ILs could have potential applications as general antimicrobials, the minimal inhibitory concentration (MIC) and minimal bactericidal concentration (MBC) of all 12 morpholinium-based HILs were determined with a set 2 Gram-positive (*L. monocytogenes* and *L. lactis*) as well as 3 Gram-negative (*E. coli*, *S. enterica* and *P. syringiae*) bacterial species.

**Figure 3:**
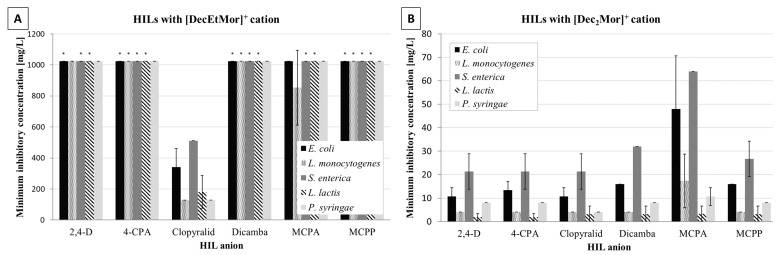
Mean minimum inhibitory concentration MIC [mg/L] ± SD of HILs with either [DecEtMor]^+^ cation (**A**) or [Dec_2_Mor]^+^ cation (**B**) for bacterial hosts: *E*. *coli*, *L. monocytogenes*, *S. enterica*, *L*. *lactis*, *P. syringae*; * No antimicrobial effect observed at 1024 mg/L concentration. Note the ordinate scale differences between the two panels.

Evaluation of the antimicrobial activity of the 12 morpholinium-based HILs showed considerable differences between the ILs concerning their chemical structure but only limited differences in regard to the tested species. Ionic liquids containing the [DecEtMor]+ cation showed limited antibacterial activity (Figure 3A). Solely IL [DecEtMor][Clopyralid] inhibited the growth of all tested bacterial species in the range 128 (*L. monocytogenes* and *P. syringae*), 181 mg/L for *L. lactis*, 341 for *E. coli* and 512 for *S. enterica*, while the other five [DecEtMor] based ILs were not able to inhibit the growth of all five bacterial hosts. In contrast, ionic liquids containing the [Dec_2_Mor]^+^ cation demonstrated high antibacterial activity against all five bacterial species in the range from 2 to 48 mg/L (Figure 3B), with *S. enterica* being more resistant than other species. The results also showed that the antimicrobial effect of the morpholinium-based HILs is not limited to growth inhibition of the tested species but that the ILs were actually biocidal in the same concentration range (Figure S1).

### 2.3. Virucidal Toxicity

To determine the potential virucidal activity of morpholinium-based HILs, five viruses with different structural characteristics as well as relevance in the human and food industry were chosen as targets. Based on the studies of Fister et al. (2017) [35], Sommer et al. (2018) [8], Gundolf et al. (2019) [9] and Bromberger et al. (2020) [10], we investigated the bacterial viruses P100, MS2 and Phi6. In addition to these viruses, the virus set-up was enlarged to gain detailed information on whether viruses with similar structures also show similar behavior. Thus, viruses P001 and PRD1 were added due to their relevance either in the food industry or in human medicine. In general, the phages P100 and P001 were investigated due to their huge structural similarity, as both are tailed bacteriophages. Furthermore, both viruses were chosen due to their relevance in the food industry. While P100 is used as a beneficial tool against the bacterial pathogen *L. monocytogenes* [26], the *Lactococcus* spp., phage P001 was selected as a representative for lactic acid bacteria (LAB) phages, that are known as the major cause of fermentation failures in the dairy industry [28,36]. Besides the bacteriophages relevant in the food industry, also phages that are used as surrogates for human pathogenic viruses are included. Therefore, we selected the bacteriophages PRD1, MS2 and Phi6. Phages PRD1 and MS2 are known as representatives for small non-enveloped viruses and enteric viruses such as adenoviruses [37], rotaviruses [38] and noroviruses [39]. In particular, phage Phi6 holds increased interest these days, as it is also increasingly used as a surrogate for enveloped human pathogenic viruses, such as influenza [40], ebola virus and coronaviruses [41]. For each of the twelve morpholinium-based HILs, the minimal virucidal concentration (VC) defined by a >4 Log reduction of infectious virus titre after treatment was determined for each of the five viruses, and the results are shown in Table 1. 

In the case of the non-enveloped phages MS2 and PRD1, none of the investigated ILs led to a >4 Log reduction of the virus titre at the highest tested concentration of 50,000 mg/L. In the case of P100, also a non-enveloped phage, virucidal activity was determined for two ILs [Dec_2_Mor][Clopyralid] (VC: 27,000 mg/L) and [DecEtMor][Dicamba] (VC: 17,286 mg/L), while the other 10 HILs were also ineffective. Although phage P001 is also a tailed virus, like phage P100, increased inactivation behavior could be determined with all liquids. While the ILs with a [DecEtMor] cation indicated a virucidal effect at 10,000 mg/L, ILs with a [Dec_2_Mor] cation exhibited enhanced inactivation with a VC at 1000 mg/L. The most sensitive particle in this set-up was the enveloped phage Phi6, which indicated virucidal inactivation at concentrations down to 70 mg/L. Similar to the results of phage P001, the ILs with a [DecEtMor] cation were less effective as HILs with the [Dec_2_Mor] cation. Five out of six [DecEtMor]-ILs exhibited a VC at 10,000 mg/L and [DecEtMor][Clopyralid] indicated a VC of 1000 mg/L. The [Dec_2_Mor]-ILs turned out to be the most effective ones for phage Phi6, as inactivation of more than 4 log units could be achieved at a concentration of 100 mg/L and less.

## 3. Discussion

The aim of this study was to determine the antibacterial and virucidal potential of twelve morpholinium-based HILs by using a recently established tripartite biological test system for IL toxicity and to evaluate if these novel ILs represent a promising new class of antimicrobials or even virucides. 

### 3.1. Enzyme Inhibition and Antimicrobial Activity

Enzyme inhibition assay is considered a good predictor of overall ILs toxicity, and it is widely used for the assessment of the environmental safety of ionic liquids. The methods most commonly referred to are based on acetylcholinesterase (AChE) [42] and DNA-polymerase enzymes [9], but also AMP deaminase [43], monooxygenase P450 [44] or elastase [45] based assays have been used.

In the case of ionic liquids, previous studies reported different structure–activity relationships for their inhibitory potential. Results obtained from acetylcholinesterase assays performed on a wide set of ionic liquids indicated a great importance of cationic head group for ILs inhibitory activity. According to those results, ILs bearing quinolonium, pyridinium, and piperidinium cation were found to be the most effective inhibitors of AChE, while phosphonium- and morpholinium-based ionic liquids were the least effective [42,46,47,48]. The lower inhibitory potential of morpholinium cation was to be explained by its lack of aromaticity and therefore lower lipohilicity [47]. Indeed, we observed that ionic liquids with [DecEtMor]^+^ morpholinium cation showed low or no inhibition of DNA polymerase with respect to their MIC values and low inhibition regarding their EC_50_ values. At the same time, ionic liquids with [Dec_2_Mor]^+^ cation were found to be a potent inhibitor of DNA polymerase. That is because another strong structural determinant of ionic liquids activity for enzyme inhibition is the length of alkyl side chain. Similar to the toxicity toward microorganisms, the inhibitory activity of ionic liquids increases with increasing the length of alkyl side chains [10,49], but also with increasing the number of long alkyl groups [8]. Since the morpholine ring of the [Dec_2_Mor]^+^ cation possess two long decyl substituents, whereas there is only one such group in the [DecEtMor]^+^ cation, the results obtained in this study fit very well with previously reported observations. 

It also has been previously reported that the anionic moiety can influence the inhibitory potential of ionic liquids. In the case of fatty acid-based ILs, longer alkyl chains of anions cause stronger inhibition of DNA polymerase enzyme, in a manner similar to the one observed for increasing the length of the cations side chain [9]. On the other hand, smaller inorganic anions can also affect enzyme inhibition activity of ionic liquids, e.g., according to their increasing chaotropicity [8]. However, it does not always follow the order of Hofmeister series [42,44]. In our study, a minor effect of anion could be observed as well. Among ionic liquids having [DecEtMor]^+^ cation only, [DecEtMor][Clopyralid] was able to completely inhibit the DNA polymerase action. Thus, the observed inhibitory effect can be assigned to the action of the clopyralid anion itself. Clopyralid is an herbicide from the picolinic acid family. To date, there are no reports on the clopyralid effect on DNA polymerase activity; however, it is known that clopyralid is able to inhibit some enzymes, such as the one involved in denitrification process [50].

The antimicrobial activity of all twelve morpholinium-based HILs on the five bacterial strains show that ionic liquids with [DecEtMor]^+^ cation exhibit no toxicity toward the bacteria tested, contrary to the compounds with [Dec_2_Mor]^+^ moiety that are able to efficiently inhibit bacterial growth below the highest tested concentration of 1000 mg/L. The only exception is [DecEtMor][Clopyralid] that showed antimicrobial activity toward all tested species at concentrations equal to 128 mg/L for *L*. *monocytogenes* and *P*. *syringae*, 181 mg/L for *L*. *lactis*, 341 mg/L for *E*. *coli* and 512 mg/L for *S*. *enterica*; however, the toxicity of this ionic liquid was far less pronounced compared to the corresponding [Dec_2_Mor][Clopyralid] compound. The differences in antimicrobial potency observed for tested ILs are due to the cation structure involving the length of alkyl chains used as substituents. While the [Dec_2_Mor]^+^ cation possesses two decyl groups bound to the central morpholine ring, [DecEtMor]^+^ ion has only one such group accompanied with another ethyl group. The same was observed by Cłapa et al. (2021) during the investigation of four *P. aeruginosa* strains, including three clinical isolates [5]. Moreover, the obtained results are in line with the trend described in numerous studies, indicating that the increased length of alkyl substituents and the number of long alkyl groups is one of the strongest determinants of ionic liquids toxicity against bacteria [6,15,51,52,53].

Notable differences in resistance level were also observed between the bacterial species tested. Among all examined strains, *S. enterica* proved to be the species most resistant to ILs action, being characterized by MIC values 8 to 14 times higher compared to *L. lactis*, the most susceptible bacterial species tested. Such or even larger disparities in susceptibility levels found between different strains are not unusual [11,54,55]. Cłapa et al. reported that of four *P. aeruginosa* strains challenged by the morpholinium ionic liquids described here, one was found fully resistant to the ILs action [5]. It proves that inter-species differences in resistance level marked in this study are not abnormal since even greater intra-species variability could be observed. Based on numerous studies on ILs interaction with microorganisms, a general trend of increased resistance of Gram-negative bacteria compared to Gram-positive species was noted [54,56]. The same tendency was observed in this study as well. The two most susceptible strains were Gram-positive *L. lactis* and *L. monocytogenes*, while other examined Gram-negative species turned out to be more resistant to the ILs tested. In the study by Weyhing-Zerrer et al. (2017), 12 bacteria species were challenged by imidazolium- and quaternary ammonium-based ILs [11]. Among those four strains were the same as those used here: *E. coli*, *L. lactis*, *L. monocytogenes* and *S. enterica*. Similar to our results, *L. lactis* was found to be the least resistant strain, far more susceptible to ILs action than *S. enterica* and *E. coli* [11]. Thus, it indicates that species-dependent toxicity patterns do not differ between morpholinium ionic liquids and imidazolium- or quaternary ammonium-based ILs. 

Overall, the results of the enzyme inhibition assay and bacterial growth inhibition tests are in great agreement regarding the structures of the compounds tested. Thus, the DNA polymerase inhibition assay was proved to be a useful tool for predicting the antimicrobial activity of ionic liquids.

### 3.2. Virucidal Activity of Morpholinium Based HILs

Morpholinium-based HILs were characterized by varying activity against the bacteriophage species tested. Out of all bacteriophages examined, the P001 and Phi6 phages titre was reduced by all ionic liquids, with the latter being the most susceptible out of all species. The P100 phage responded only to [DecEtMor][Dicamba] and [Dec_2_Mor][Clopyralid] compounds, whereas PRD1 and MS2 bacteriophages turned out to be completely resistant to all ionic liquids. 

The least resistant Phi6 phage was the only enveloped virus among all species tested. Contrary to non-enveloped viruses, the nucleocapsid of enveloped viruses is surrounded by a phospholipid bilayer containing proteins and glycoproteins [57]. Enveloped viruses are less persistent than non-enveloped viruses [58]. Additionally, because the viral envelope can be targeted by detergents, the enveloped viruses are generally found to be more prone to surfactants [59]. Lipid membranes are also one of the main sites of action for ionic liquids. The toxicity of ILs may be a result of their sorption on membrane or due to bilayer disruption [12,60]. Moreover, the introduction of longer hydrophobic alkyl chains to the ILs structure enhances this effect, stimulating membrane disruption [61]. We observed a higher virucidal activity of ionic liquids bearing more hydrophobic cation against Phi6. Such an effect has been already reported for Phi6 bacteriophage in numerous studies before [8,9,10]. Therefore, it can be concluded that the increased vulnerability of the Phi6 phage toward ionic liquids and the marked “side chain” effect can be explained primarily by the presence of the viral envelope.

*Lactococcus* spp. phage P001 was inactivated by all ILs tested. P001 belongs to morphology-based family *Siphoviridae* involving non-enveloped, tailed bacteriophages with a long non-contractile tails [62]. P001 also represents lactic acid bacteria (LAB) phages, which pose a major threat in dairy industries [28,36]. For the first time, the ability of ionic liquids to effectively control phages that hinder the production of dairy products was described. Since the net charge of bacteriophages from *Siphoviridae* family is negative, the obtained results could be explained by electrostatic interactions between morpholinium cations and virus surface [63,64,65]. While a clear effect of the number of long side chains on virulence was also observed for P001, ionic interactions cannot be the only factor involved. It was demonstrated before by Ly-Chatain et al. (2013) that c2 phage, a LAB phage closely related to P001, can be controlled by chitosan and CTAB [66]. Although the decrease in virus titres observed by the authors was mainly due to electrostatic interactions, CTAB cations with a long hexadecyl alkyl chain (16 C) were found to be far more effective against the c2 phage compared to the hydrophilic chitosan cations. This corresponds well with the increased activity of [Dec_2_Mor] ILs and suggests that cation hydrophobicity could play an important role in the inactivation of bacteriophages. That said, no cation effect could be observed for any of the three remaining phages: MS2, PRD1 or P100.

In previous studies, the P100 phage was found to be inhibited by imidazolium- and ammonium-based ionic liquids with side chains composed of 10–14 carbon atoms [8,10,35]. Moreover, ILs with two long substituents in their structure exhibited higher virucidal potential against the described phage. We were unable to confirm this observation since neither [Dec_2_Mor] nor [DecEtMor] ILs could inactivate P100 particles (except [Dec_2_Mor][Clopyralid] and [DecEtMor][Dicamba]). That difference could be due to the lesser biological activity of cations with a morpholinium head group compared to the quaternary ammonium or imidazolium groups. On the other hand, virucidal activity observed for [Dec_2_Mor][Clopyralid] and [DecEtMor][Dicamba] compounds could be influenced by the corresponding anions. The role of anions in phage inactivation has already been noted. For example, it was demonstrated that ionic liquids with long-chain fatty acid anions exhibit high virucidal activity against P100 phage [9]. Moreover, Fister et al. (2017) investigated the effect of a wide range of anions in ionic liquids formulation on the infectivity of the P100 phage [35]. The authors have shown the high inactivation rate for ILs with salicylate anion, a derivative of benzoate anion. In contrast, ILs having just benzoate anion exhibited very weak activity against the P100 phage. Interestingly, dicamba is also a benzoate anion derivative, and the antiviral activity of benzoic acid or salts of its derivatives, such as propyl 4-hydroxybenzoate, has previously been demonstrated against representatives of tailed bacteriophages [67]. That indicates that [DecEtMor][Dicamba] activity can be due to the structure of the dicamba anion. Clopyralid, having a pyridine ring, belongs to picolinic acid family. Compounds with pyridine cations, such as cetylpyridinium chloride or pyridinium formate, can inactivate tailed phages and human viruses [68,69,70]. Additionally, picolinic acid has been proven as a virucidal agent against human viruses, such as CHIKV, VZV or SARS-CoV-2 [71,72,73]. On top of that, the increased activity of [DecEtMor][Clopyralid] compared to the ILs with the same cation was observed toward P001 and Phi6 phages as well. Those results highlight the role of clopyralid anion in the modulation of ILs toxicity against viruses. That said, since no increased activity against P100 phage could be noted for clopyralid paired with [DecEtMor]^+^ cation nor for dicamba paired with the [Dec_2_Mor]^+^ cation, the obtained results cannot be explained by the action of anion alone.

The differences in the resistance level between P100 phage and P001 phage are also noteworthy. P100 is a non-enveloped tailed virus that belongs to the same order as P001 phage. However, since it possesses a long contractile tail, which is morphologically distinct from P001 phage tail, P100 is classified as the morphology-based family *Myoviridae*. Up to date, no studies have been conducted to compare the effects of ionic liquids on different morphological types of tailed viruses. Myophages are reported to be robust against photocatalytic treatment, surfactants or biocides such as ethanol or sodium hypochlorite, even when compared with other tailed viruses [30,74,75]. Brown et al. (2020) have shown that KOX1 phage representing *Myoviridae* was far more resistant against all preservatives examined (benzoic acid, chlorocresol or 2-phenoxyethanol included), compared to the member of *Siphoviridae*—PAC1 phage [67]. Thus, myoviruses can be found to be more resistant than siphoviruses toward some antiviral agents. On top of that, the obtained results could be explained by differences in the tail morphology of P100 and P001 phages. While the tails of *Siphoviridae* members are non-contractile and flexible, myophages tails are contractile and rigid. Additionally, the central tail tube of myophages is surrounded by protein tail sheath [76]. Just like siphophages, the surface of *Myoviridae* members has a net negative charge [64,77,78,79]. Richter et al. (2021) have shown that nanoparticles coated with positively charged, long-chained TMA molecules (*N*,*N*,*N*-trimethyl(11-mercaptoundecyl)ammonium) can bind to the tail of T4 myovirus, leading to the inactivation of the virus. At the same time, the representative of siphophages T1 phage was more susceptible to TMA nanoparticles than the T4 virus [78]. Considering the structural similarities between TMA molecules and the cations of ILs used in this study, and the higher sensitivity of the siphovirus observed in both cases, we can hypothesize that morpholinium cations may act in a similar manner to TMA molecules through electrostatic interactions with bacteriophage tails. In view of the above, the presence of a tail sheath may protect the myoviruses from inactivation to some extent, making P100 particles more resistant to ILs compared to the P001 phages with no tail sheath. Although this seems to be a reasonable explanation for the differences observed, this hypothesis needs further investigation. 

Both MS2 and PRD1 phages were insensitive to all ILs tested. The lack of inactivation of MS2 phage by morpholinium-based ILs is in good accordance with data collected for other types of ionic liquids. The extremely high robustness of MS2 phage has been described before for imidazolium- and quaternaryammonium-based ionic liquids, pyrithione-based ionic liquids and ionic liquids with fatty acid anions [8,10,35,36]. To date, no data were available for ILs effect on PRD1 bacteriophage. MS2 is a small isometric phage, with a diameter of about 25 nm [80]. Due to its small size and structural simplicity, the number of target sites for ILs action is quite limited. PRD1 is also an isometric bacteriophage, twice the MS2 diameter size. Its particle is composed of a lipid membrane surrounded by protein capsid [81]. Since the lipid vesicle is located in the interior capsid, it cannot be directly targeted by ionic liquids. Both phages are reported to be eradicated by commonly used disinfectants and household chemicals like H_2_O_2_, trisodium phosphate or dishwashing detergents [82]. At the same time, both phages were demonstrated to be more persistent to UV light compared to enveloped and tailed phages [83,84]. Additionally, PRD1 showed very high persistence in ultrapure water subjected to different kind of treatments [85] and was found extremely robust against a number of nanoparticle–halogen adducts and iodine treatment [86,87]. Additionally, MS2 was reported to be more resistant to CTAB surfactant or TMA nanoparticles compared to tailed phages [66,78]. It is known that the aggregation of viruses is one of viral resistance mechanisms against disinfectants [88,89]. There are reports that MS2 phages can aggregate, which leads to a decrease in biocides inactivation efficacy [90,91]. The same process was noted for PRD1 as well [92]. Thus, the aggregation ability might have contributed to the increased resistance against ionic liquids displayed by examined phages.

## 4. Materials and Methods

### 4.1. Ionic Liquids

For the present study, twelve morpholinium ionic liquids (Ils) were selected. Six of them consisted of a 4,4-didecylmorpholinium [Dec_2_Mor]^+^ cation and another six of 4-decyl-4-ethylmorpholinium [DecEtMor]^+^ cation with one of the following anions: 2,4-dichlorophenoxyacetate [2,4-D]^−^; 4-chloro-2-methylphenoxyacetate [MCPA]^−^; 3,6-dichloro-2-methoxybenzoate [Dicamba]^−^; (±)-2-(4-chloro-2-methylphenoxy)propionate [MCPP]^−^; 4-chlorophenoxyacetate [4-CPA]^−^; or 3,6-dichloro-2-pyridinecarboxylate [Clopyralid]^−^ (Table S1). The synthesis and characterization of ionic liquids was presented earlier [5,93].

### 4.2. Antibacterial Activity Assays

The determination of the ILs minimum inhibitory concentration (MIC) was performed for all virus hosts on 96-well plates. Firstly, 80 µL of test compounds was distributed to each well in the top row of the plate at a concentration equal to 5120 mg/L. Next, 40 µL of sterile TSB-Y medium was added to empty wells and serial dilution was carried out. Then, bacterial suspensions were prepared. First, TSB-Y broth was inoculated with overnight cultures of investigated bacterial strains to a final concentration of 5 × 10^5^ CFU/mL. Such prepared inoculums were distributed to all wells in a volume of 160 µL. Plates were incubated overnight at a temperature adequate for the growth of investigated strain. Bacterial growth was marked by a change of medium turbidity. MIC values were recognized as the lowest concentration for which no turbidity change could be observed.

After MIC determination, the minimum bactericidal concentration (MBC) was evaluated. Briefly, 10 µL aliquots of cultures was collected from all wells where no bacterial growth was observed and then transferred on TSA-Y plates. Agar plates were incubated overnight at a temperature adequate for the growth of investigated strain. The lowest concentration of test compound for which no bacterial colonies were observed was identified as MBC.

### 4.3. Enzyme Inhibition Assay

To determine the toxicological potential of ILs on enzymatic level a qPCR enzyme inhibition assay was performed according to a previously published protocol [9]. Enzymatic inhibition was defined as the half-maximum effective concentration (EC50 value), determined by linear regression resulting in a 50% efficiency of the qPCR reaction. Besides, the minimal inhibitory concentration (MIC) as the lowest concentration, which prevents enzymatic activity was determined. One qPCR reaction of 25 μL final volume contained 2.5 μL of 10× reaction buffer (Biozym Scientific GmbH, Hessisch Oldendorf, Germany), 500 nM of each primer (*LIP1*: 5′-GAT ACA GAA ACA TCG GTT GGC-3′ and *LIP2*: 5′-GTG TAA TCT TGA TGC CAT CAG G-3′ (both Eurofins Genomics, Ebersberg, Germany), 1.25 μM EvaGreen^®^, 200 μM of each dNTPs (dATP, dTTP, dGTP and dCTP), 1 U of Taq DNA Polymerase (Biozym Scientific GmbH, Hessisch Oldendorf, Germany), ~150 copies of the internal amplification control *L. monocytogenes* EGDe and 5 μL of the respective IL solution. Real-time PCR was performed in an Mx3000p real-time PCR thermocycler (Stratagene, San Diego, CA, USA), where amplification followed an internal denaturation at 94 °C for 5 min for 45 cycles at 94 °C for 15 s and 64 °C for 1 min. A final extension was performed for 1 min at 95 °C, 30 s at 55 °C and 30 s at 95 °C. Each qPCR experiment was performed at least three times on different days, with 5% (50,000 mg/L) as the highest tested concentration. 

### 4.4. Viruses and Related Host Strains

Five bacteriophages, P100 (non-enveloped), P001 (non-enveloped), PRD1 (non-enveloped), MS2 (non-enveloped) and Phi6 (enveloped), were used in this study. Virus P100 was purchased as Listex ™ P100 solution (Batch 12G26, Lot: 308; Micreos, Wageningen, The Netherlands). Virus MS2 was kindly provided by Regina Sommer, Medical University of Vienna. Virus Phi6 was purchased from the German Collection of Microorganisms and Cell Cultures (DSMZ, Braunschweig, Germany; DSM No. 21518). Virus PRD1 was purchased from the German Collection of Microorganisms and Cell Cultures (DSMZ, Braunschweig, Germany; DSM No. 19107). Virus P001 was purchased from the German Collection of Microorganisms and Cell Cultures (DSMZ, Braunschweig, Germany; DSM No. 4262).

The viruses were chosen, on one hand, according to some special structural characteristics, such as size, envelope structure and genome structure shown in Table 2. On the other hand, the bacteriophages were chosen based on their relevance in the food industry and human medicine, whereas phages P100 and P001 are of special interest in the food industry, while the phages PRD1, MS2 and Phi6 are used in human medicine as viral surrogates for human pathogenic viruses. 

The bacterial host strains for the respective viruses were cultured overnight in tryptone soya broth (TSB) with 0.6% (*w*/*v*) yeast extract (Oxoid Ltd., Hampshire, UK). *Listeria monocytogenes* EGDe (ATCC BAA-679) used for phage P100, *Salmonella enterica* used for PRD1 (DSMZ, Braunschweig, Germany; DSM No. 19207) and *Escherichia coli* Top 10F′ (Thermo Fisher Scientific, Waltham, MA, USA) used for phage MS2 were incubated overnight at 37 °C, whereas *Lactococcus lactis* (DSMZ, DSM No. 4366) used for phage P001 and *Pseudomonas syringae* (DSMZ, DSM No. 21482) used for phage Phi6 was incubated overnight at 25–27 °C. Overnight cultures of *E. coli* Top 10F′ were diluted 10-fold in fresh medium and incubated at 37 °C for 3 to 6 h to provide a maximum number of viable cells in the logarithmic growth phase (log phase). Virus stock solutions (P100, P001, PRD1, MS2, and Phi6) were used for plaque assays and plates with confluent lyse were used. These plates were overlaid with 5 mL saline magnesium (SM) buffer (5.8 g of NaCl, 2.4 g of Tris-HCl, 1.0 g of CaCl_2_, 0.1 g of gelatine, and 1 L of H_2_O; pH 7.5) described by Kropinski et al. (2009) and shaken overnight at 4 °C [95]. Afterwards, the SM buffer was separated and centrifuged at 8000 rpm for 2 min. The supernatant was filtered (0.02 μm), aliquoted, and stored at −20 °C. 

### 4.5. Numeration of Plaque Forming Units

All viruses were used at concentrations of approximately 10^9^–10^11^ plaque forming units/mL (PFU/mL). The virus titre of phage P100, P001, PRD1 and Phi6 was determined using the Small Drop Plaque Assay [96]. In short, an overnight culture of the respective bacterial strain was 10-fold diluted in SM buffer [95,96]. Afterwards, a 10-fold serial dilution of the viruses was prepared in the respective bacteria-containing buffer. After an incubation time of the respective host-related temperature, 20 μL of each dilution were dropped on a tryptone soya agar (TSA)-plate and incubated overnight at 37 °C or 25–27 °C. After 16 h of incubation, plaques were counted and compared to the untreated positive controls. Besides the Small Drop Plaque Assay, the Double Agar Overlay Assay was used for the numeration of the bacteriophage MS2 [96]. Therefore a 10-fold dilution series of the MS2 was prepared in SM buffer and afterwards an equal volume of log phase culture of *E. coli* Top 10F′ (OD_600_ = 0.6–0.8) was added to each virus dilution. Finally, 200 μL of the phage–bacteria mixture was mixed with 3 mL prewarmed overlay medium (TSB with 0.3% agar) and spread on a TSA-plate. The plates were incubated at the respective bacterial host-related temperature for 16 h as soon as the overlay medium was hardened. After, the overnight incubation plaques were counted and compared to the untreated positive controls.

### 4.6. Virucidal Concentration (VC)

All viruses were mixed with each IL—solution to reach a final concentration of 1–50,000 mg/L (*w*/*v* in water) and were incubated for 30 min at room temperature to determine the minimum virucidal concentration (VC). Afterwards, the log_10_ reduction of virus titre (plaque-forming units (PFU)/mL), in comparison to the untreated positive control, was calculated. All ILs were initially tested at a concentration of 50,000 mg/L or 25,000 mg/L (*w*/*v* in water), whereas ILs, which caused a reduction of ≥4 log_10_ units or reached the detection limit of the assay were additionally tested down to concentrations of 25,000 mg/L, 10,000 mg/L and 1000 mg/L, 100 mg/L, 10 mg/L and 1 mg/L until the VC was reached. The VC was defined as the lowest concentration of the respective ionic liquids where the virus titre decreased by equal or greater than 4 log_10_ units or the reach of the detection limit, which is also equated with a reduction of at least 4 log_10_ units. All experiments included positive as well as negative controls and were performed in duplicate on at least two different days or individually on three different days. 

## 5. Conclusions

In this study, the antimicrobial and virucidal activity of morpholinium based HILs was determined to evaluate if such ILs can serve as a promising new class of novel biocides. The results showed a higher activity of [Dec_2_Mor]^+^ based HILs compared to [DecEtMor]^+^ based HILs, while the effect of the herbicidal anions was limited. Overall, it was successfully demonstrated that [Dec_2_Mor]^+^ based HILs can be considered effective antimicrobials against a broad spectrum of bacterial species. The virucidal tests showed that ILs antiviral activity depends on the type and structure of the virus, revealing enveloped Phi6 phage as being highly susceptible to the ILs action, while the non-enveloped phages PRD1 and MS2 proved completely resistant to ionic liquids. Furthermore, a comparison of results obtained for P100 and P001 phages demonstrated for the first time that the susceptibility of viruses to ionic liquids could be dependent on differences in the phage tail structure.

The fact that only limited effects of the HILs anions was found on all three biological test systems, also demonstrates that there is possible room for further improving biocidal activity by incorporation of either antimicrobial or virucidal anions in the future. 

## Figures and Tables

**Table 1:** Virucidal concentration [mg/L] (including upper and lower limits) of the 12 ILs.

Virucidal Concentration mg/L					
Ionic Liquids	P100	P001	PRD1	MS2	Phi6
[Dec_2_Mor][2,4-D]	>50,000 ^1^	1000 ^2^	> 50,000 ^1^	>50,000 ^1^	100 ^3^
[Dec_2_Mor][4-CPA]	>50,000 ^1^	1000 ^2^	> 50,000 ^1^	>50,000 ^1^	100 ^3^
[Dec_2_Mor][Clopyralid]	27,000	1000 ^2^	> 50,000 ^1^	>50,000 ^1^	100 ^3^
[Dec_2_Mor][Dicamba]	>50,000 ^1^	1000 ^2^	> 50,000 ^1^	>50,000 ^1^	100 ^3^
[Dec_2_Mor][MCPA]	>50,000 ^1^	1000 ^2^	> 25,000 *	>50,000 ^1^	100 ^3^
[Dec_2_Mor][MCPP]	>50,000 ^1^	1000 ^2^	> 50,000 ^1^	>50,000 ^1^	70 ^4^
[DecEtMor][2,4-D]	>50,000 ^1^	10,000 ^5^	> 50,000 ^1^	>50,000 ^1^	10,000 ^5^
[DecEtMor][4-CPA]	>50,000 ^1^	10,000 ^5^	> 50,000 ^1^	>50,000 ^1^	10,000 ^5^
[DecEtMor][Clopyralid]	>50,000 ^1^	1000 ^2^	> 50,000 ^1^	>50,000 ^1^	1000 ^2^
[DecEtMor][Dicamba]	17,286 ^6^	10,000 ^5^	> 50,000 ^1^	>50,000 ^1^	10,000 ^5^
[DecEtMor][MCPA]	>50,000 ^1^	10,000 ^5^	> 50,000 ^1^	>50,000 ^1^	10,000 ^5^
[DecEtMor][MCPP]	>50,000 ^1^	10,000 ^5^	> 50,000 ^1^	>50,000 ^1^	10,000 ^5^

Upper and lower limits: ^1^ (>50,000; >50,000); ^2^ (1000; 1000); ^3^ (100; 100); ^4^ (100; 10); ^5^ (10,000; 10,000); ^6^ (25,000; 1000). * Preparation of an aqueous solution of >25,000 mg/L was not possible.

**Table 2:** Virus characterization and their hosts.

	P100	P001	PRD1	MS2	Phi6
Envelope	No	No	No	No	Yes
Genome	DNA	DNA	DNA	RNA	RNA
Genome size	130–160 kb (137 kb)	Ca. 50 kb	15 kb	3.4–4.3 kb	13.3 kb
DS/SS	ds	ds	ds	+ss	ds
Family	*Herelleviridae*^a^ (Formerly *Myoviridae*)	Unclassified ^a^ (Formerly *Siphoviridae*)	*Tectiviridae*	*Fiersviridae*^a^ (Formerly *Leviviridae*)	*Cystoviridae*
Surrogate	-	LAB-phages	*Adenoviruse*, *Rotaviruses*	*Enteric Viruses*, *Norovirus*	*Ebola*, *Influenza*, *Corona*
Host	*Listeria monocytogenes*	*Lactococcus lactis*	*Salmonella enterica*	*Escherichia coli* (Top 10 F′)	*Pseudomonas syringae*

^a^ Nomenclature according to the newest virus taxonomy approved and ratified by the International Committee on Taxonomy of Viruses (ICTV) in March 2022 [94].

## Data Availability

Not applicable.

## Supplementary Materials

The following supporting information can be downloaded at: https://www.mdpi.com/article/10.3390/ijms24021686/s1.
