# Supplementary material for: Antimicrobial and Virucidal Potential of Morpholinium-Based Ionic Liquids
Source: Int J Mol Sci. 2023 Jan 14;24(2):1686. doi: 10.3390/ijms24021686 (PMC9863300; doi:10.3390/ijms24021686)
Supplement: Supplementary file 1 [file ijms-24-01686-s001.zip › ijms-2120778-supplementary.pdf]

International Journal of *Molecular Sciences*

MDPI

Supplementary Materials

Antimicrobial and virucidal potential of morpholinium-based ionic liquids

Jakub Michalski [1], Julia Sommer [2,3], Peter Rossmanith [2,4], Patrick Mester [2,4], Anna Syguda [5] and Tomasz Clapa [1,*]

[1] Poznań University of Life Sciences, Department of Biochemistry and Biotechnology, Dojazd 11, 60-632 Poznan, Poland; jak.michalski@onet.pl
[2] Christian Doppler Laboratory for Monitoring of Microbial Contaminants, Unit for Food Microbiology, Department of Veterinary Public Health and Food Science, University of Veterinary Medicine, Vienna, Austria; peter.rossmanith@vetmeduni.ac.at
[3] Epitome GmbH, The ICON Vienna, Tower 17, Gertrude-Fröhlich-Sandner-Str. 2-4 ,1100 Vienna, Austria; julia.sommer@epitome.inc
[4] Unit of Food Microbiology, Institute of Food Safety, Food Technology and Veterinary Public Health Department for Farm Animals and Veterinary Public Health University of Veterinary Medicine Vienna, Veterinärplatz 1, 1210 Vienna, Austria; Patrick-julian.mester@vetmeduni.ac.at
[5] Poznan University of Technology, Department of Chemical Technology, Berdychowo 4, 60-965 Poznan, Poland; anna.syguda@put.poznan.pl;
* Correspondence: tomasz.clapa@up.poznan.pl

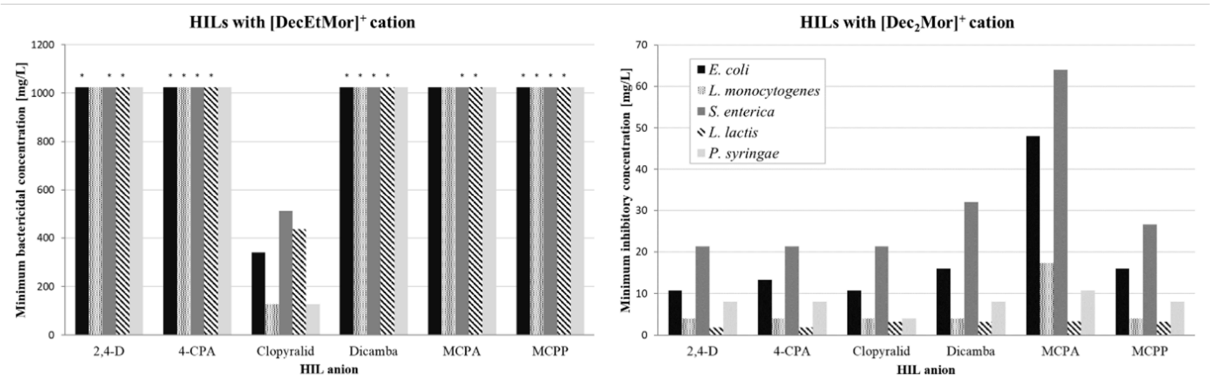

Figure S1. Mean bactericidal inhibitory concentration MBC [mg/L] of HILs with either [DecEtMor]+ cation (**A**) or [Dec2Mor]+ cation (**B**) for bacterial hosts: *E. coli*, *L. monocytogenes*, *S. enterica*, *L. lactis*, *P. syringae*; * No bactericidal effect observed at 1024 mg/L concentration. Note the ordinate scale differences between the two panels.

Table S1. List of morpholinium herbicidal ionic liquids examined in the study.

Abbreviation	Acronym	Name	Chemical structure
**IL-1**	[DecEtMor] [2,4-D]	4-decyl-4-ethylmorpholinium 2,4-dichlorophenoxyacetate	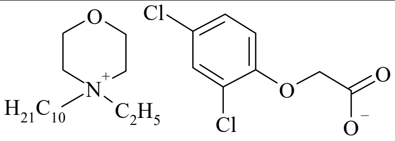
**IL-2**	[DecEtMor] [4-CPA]	4-decyl-4-ethylmorpholinium 4-chlorophenoxyacetate	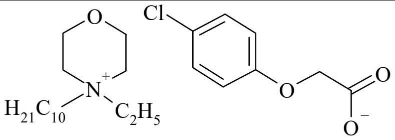
**IL-3**	[DecEtMor] [Clopyralid]	4-decyl-4-ethylmorpholinium 3,6-dichloro-2-pyridinecarboxylate	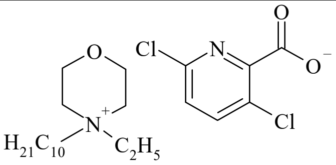
**IL-4**	[DecEtMor] [Dicamba]	4-decyl-4-ethylmorpholinium 3,6-dichloro-2-methoxybenzoate	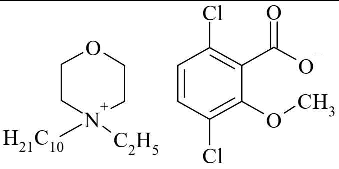
**IL-5**	[DecEtMor] [MCPA]	4-decyl-4-ethylmorpholinium 4-chloro-2-methylphenoxyacetate	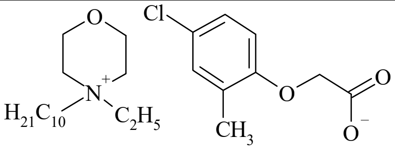
**IL-6**	[DecEtMor] [MCPP]	4-decyl-4-ethylmorpholinium (±)-2-(4-chloro-2-methylphenoxy)propionate	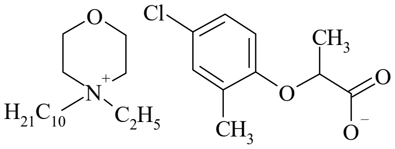
**IL-7**	[Dec$_2$Mor] [2,4-D]	4,4-didecylmorpholinium 2,4-dichlorophenoxyacetate	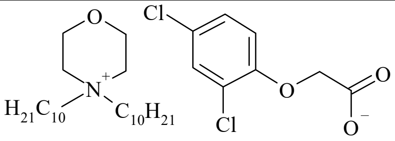
**IL-8**	[Dec$_2$Mor] [4-CPA]	4,4-didecylmorpholinium 4-chlorophenoxyacetate	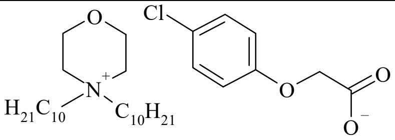
**IL-9**	[Dec$_2$Mor] [Clopyralid]	4,4-didecylmorpholinium 3,6-dichloro-2-pyridinecarboxylate	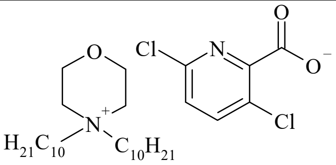
**IL-10**	[Dec$_2$Mor] [Dicamba]	4,4-didecylmorpholinium 3,6-dichloro-2-methoxybenzoate	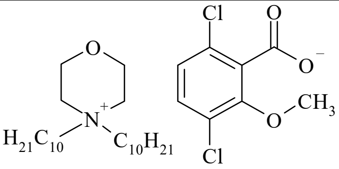
**IL-11**	[Dec$_2$Mor] [MCPA]	4,4-didecylmorpholinium 4-chloro-2-methylphenoxyacetate	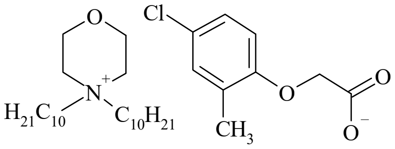

**IL-12**	[Dec2Mor] [MCPP]	4,4-didecylmorpholinium (±)-2-(4-chloro-2-methylphe-noxy)propionate	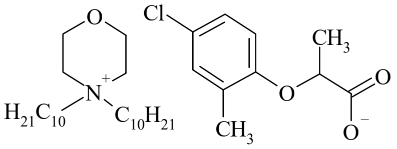

Table S2. Enzyme inhibition of HILs shown as MIC and EC_{50} [mg/L] (including upper and lower limits).

Ionic Liquids	MIC [mg/L]	EC_{50} [mg/L]
[Dec2Mor][2,4-D]	**80** (80; 80)	**21** (25,6; 16,4)
[Dec2Mor][4-CPA]	**80** (80; 80)	**10** (12,7; 6,9)
[Dec2Mor][Clopyralid]	**80** (80; 80)	**17** (30,6; 9,7)
[Dec2Mor][Dicamba]	**80** (80; 80)	**21** (37,2; 9,4)
[Dec2Mor][MCPA]	**80** (80; 80)	**24** (53,2; 9,6)
[Dec2Mor][MCPP]	**80** (80; 80)	**18** (24,9;12,2)
[DecEtMor][2,4-D]	**10.000** (10.000; 10.000)	**1034** (1347; 691)
[DecEtMor][4-CPA]	**10.000** (10.000; 10.000)	**1181** (1237; 1078)
[DecEtMor][Clopyralid]	**2000** (2000; 2000)	**751** (929; 618)
[DecEtMor][Dicamba]	**10.000** (10.000; 10.000)	**1947** (3804; 939)
[DecEtMor][MCPA]	**10.000** (10.000; 10.000)	**1120** (1162; 1052)
[DecEtMor][MCPP]	**10.000** (10.000; 10.000)	**1156** (1192; 1094)
